# Conversion of Biomass to Organic Acids by Liquefaction Reactions Under Subcritical Conditions

**DOI:** 10.3389/fchem.2020.00024

**Published:** 2020-01-29

**Authors:** Aslı Yüksel Özşen

**Affiliations:** Department of Chemical Engineering, Faculty of Engineering, Izmir Institute of Technology, Izmir, Turkey

**Keywords:** biomass, subcritical water, liquefaction, hydrolysis, electrolysis, levulinic acid

## Abstract

Recently, liquefaction of biomass in subcritical water to convert it into value-added substances has been broadly attracting attention. However, there is a gap in literature about the levulinic acid, which is a high worth substance, production from biomass using subcritical water. As a green chemistry approach, decomposition of biomass could be obtained using subcritical water effectively. In this case, water uses as a solvent so that it gives a possibility to take place a reaction for the decomposition of biomass. Subcritical water, which liquid water and its temperature is higher than the normal boiling point of water, has higher ion product as well as higher concentrations of H^+^ and OH^−^ ions. Additionally, it has high diffusivity, low viscosity and much lower dielectric constant. For instance, whereas dielectric constant of subcritical water is 80 at 298 K, it is 2 at 673 K. The point of this research paper is to assess the impacts of different reaction parameters on cellulose conversion as the principle segment of lignocellulosic biomasses for the production of value-added chemicals, particularly levulinic acid. Hazelnut shell waste was chosen as model biomass since hazelnut is a standout amongst the most cultivated agricultural crops in Turkey. Besides, Turkey provide 70% of the world's total hazelnut production. It was found that as reaction temperature increases, a considerable improvement on the amount of formed levulinic acid and conversion of hazelnut shell was observed. For instance, when the reaction temperature, time and acid concentration were 280°C, 120 min and 50 mM, respectively, levulinic acid yield and conversion of hazelnut shell were found as 13.05 and 65.40%, respectively. Addition of H_2_SO_4_ enhanced the production of levulinic acid from waste hazelnut shell. Another method which is hybrid process could be used to produce value-added chemicals from lignocellulosic biomass. Hybrid process basically combines hydrolysis and electrolysis in subcritical water. Subcritical water has much lower dielectric constant than liquid water at ambient temperature. So, it was claimed that if constant current was applied to the reaction medium through specially designed electrodes in subcritical water environment, electrolysis could alter the hydrolysis reaction of cellulose in a way of protonation of intra-and inter-molecular hydrogen bonding around anode and as a result electrolysis in subcritical water could decrease necessary thermal energy to hydrolyze the β(1–4) glycosidic linkage. Therefore, we developed a green hybrid process by combining hydrolysis and electrolysis in subcritical water without using any toxic, organic solvents and catalyst. Effects of especially applied current and temperature on the product distribution and conversions of cellulose were revealed and hydrothermal electrolysis reaction pathway of cellulose was proposed. The significance of the interaction indicated that, applied voltage had major impact on cellulose hydrolysis. Maximum cellulose conversion (82%) was achieved at 230°C and 180 min of reaction time in 25 mM of H_2_SO_4_. Application of 8.0 V of applied voltage to the reaction medium at reaction temperature of 230°C increased the TOC conversion (50.3%) with acid concentration of 25 mM in comparison with current-free experiments. Thus, the idea of electrochemically generated acid layer due to the dissociation of water around anode is supported. As future perspective, the output of the study gave an idea about converting cellulose and various biomass wastes, which may have high cellulose, content and led the way in obtaining valuable chemicals from no utilized real biomass sources such as hazelnut shell waste. The studies with other biomasses are undergoing.

## Introduction

According to BP Energy Outlook 2035 in period from 2013 to 2035 global demand of energy and CO_2_ emissions will increase by 37 and 25%, respectively. Biomass is a renewable energy source and the interest for biomass increases day by day. Since 50% of total biomass consists of plant-derived or lignocellulosic biomass and its content made-up from cellulose (38–50%), which is one of the most prevalent organic compounds in the World, hemicelluloses (23–32%), and lignin (15–25%) (Toor et al., 2011).

**Figure 1:**
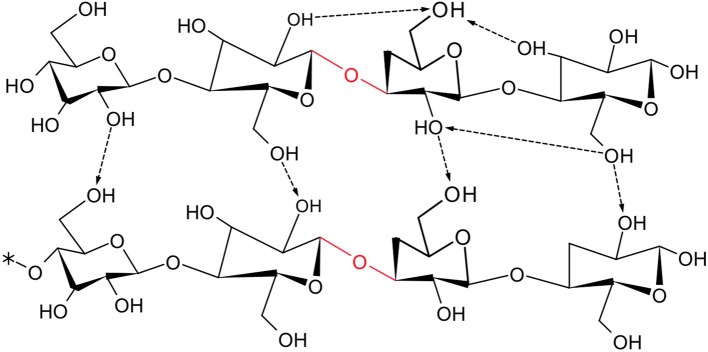
Structure of microcrystalline cellulose with ending and repeating group.

In this study, microcrystalline cellulose was used as model compound due to its crystalline structure that makes it insoluble and resistant to attack by catalyst. Cellulose has hydrogen bonds which are intra- and intermolecular, so that it has low solubility and high rigid structure (Figure 1, arrows). The challenge regarding the solvability of cellulose arises due to the intermolecular hydrogen bonds, which make intermolecular bonds inaccessible to solvent compound. Due to this structure, cellulose does not swell in water as well as it has a resistance against enzymes. However, reported studies showed that hot compressed water can rapidly solubilize cellulose and hydrolyze to its building block as glucose (Sasaki et al., 2004; Knezevic et al., 2009).

Liquefaction of cellulose in subcritical water conditions is one of the most attractive techniques for the production of high value chemicals since water has several advantages against other organic solvents. For instance, water is cheaper than other solvents and it is non-toxic, non-flammable as well as non-explosive. While water becomes a good solvent when its pressure and temperature approach its critical point, it is called as hot-pressurized water. It has a unique characteristic and its thermodynamic properties such as density, dielectric constant, viscosity, ion product, and solvating power could be tuned so that it becomes more effective on decomposition of cellulose (Savage, 1999; Yüksel, 2016).

Cellulose, a model compound, was used for the hydrothermal production of different high-value products. For instance, Saito et al. (2009) investigated the decomposition of cellulose in hot-pressurized water at 200–240°C and 15–20 MPa for 50–120 s. After 120 s, 2-furfural, 5-HMF, glycolic acid, lactic acid, and formic acid formed in high yields. Williams and Onwudili (2006) was studied about the decomposition of model compounds, which are cellulose, glucose, starch, and cassava waste, using sub- and supercritical water. These model compounds decompose to gaseous products carbon dioxide, carbon monoxide, hydrogen, and methane as well as oil and char also formed. Product distribution and gas yield was varied depending on model compound and reaction temperature. For instance, if cellulose is used as a model compound, then high yield of carbon monoxide and char is observed. Additionally, C1 and C4 hydrocarbons were formed (Yüksel, 2016). Ehara and Saka (2005) investigated the treatment of cellulose in three different systems which are sub- and supercritical water and their combined system. In each system, different yields of organics were obtained, however, precipitation was not observed in combined system. Water conditions has more effect over the reaction rate as well as decomposition mechanism of cellulose.

Levulinic acid, furfural and acetic acid are an example for bio-based chemicals and they can form by the decomposition of different types of biomass. It can be said that levulinic acid is a versatile chemical and it is in the list of Top Value-Added Chemicals from Biomass. Pharmaceutical and flavoring agents, resins, herbicides, plasticisers, anti-freeze agents, and biofuel additives can be derived from levulinic acid (Yüksel, 2016). Based on the present knowledge, firstly, hexose sugars dehydrate and the intermediate product 5-hydroxymethylfurfural (5-HMF) forms. After that, 5-HMF hydrates by the addition of water to C2–C3 bond the furan ring so that final products (levulinic and formic acids) form at the same stoichiometric ratio (Horvat et al., 1985; Asghari and Yoshida, 2010).

As model lignocellulosic biomass, waste hazelnut shell was selected since hazelnut is a considerable amount of agricultural crop in Turkey. Hazelnut produces 650,000 tons/year over the World and Turkey supplies 75% of it. During hazelnut processing, waste hazelnut shells form in large quantities and they are mainly used for heating (Guney, 2013). Hence, waste hazelnut shells could utilize to produce value-added chemicals so that they could not only be a natural alternative resource but also contribute in value gaining (Gozaydin and Yuksel, 2017).

In literature, there are several researches about the natural biomass decomposition in subcritical water. Chan et al. (2014) studied over the comparison of the decomposition of empty fruit bunch, palm mesocarp fiber and palm kernel shell by sub- and supercritical hydrothermal liquefaction. The results show that while phenolic compounds formed from palm mesocarp fiber and palm kernel shell at higher amounts, ester were only produced when palm kernel shell was used. Kruse and Gawlik (2003) investigated to description of possible reaction pathways of degradation of phytomass (mainly consist of cooked carrots and potatoes). According to the results, the degradation of HMF can be observed by two different pathways which are the formation of 1,2,4-benzenetriol and levulinic acid (via acid catalyst). Cheng et al. (2009) studied over the hydrothermal degradation of switchgrass. In this study, the highest conversion, desired product yield (5-HMF and furfural) and 90% of rapid switchgrass conversion were observed when the reaction temperature, pressure and residence time are 250–350°C, 20 MPa and 1–300 s, respectively. Asghari and Yoshida (2010) investigated the effect of dilute phosphate buffer (at pH 2) over the degradation of Japanese red pinewood in subcritical water. It was concluded that the Japanese red pine wood converted into higher amount of sugars within shorter time compared to uncatalyzed conditions. By the help of further treatment, the yield of HMF and furfural were almost doubled. The study of Tymchyshyn and Xu (2010) show that the sawdust and cornstalks was degraded into phenolic compounds at 250–350°C and 2 MPa H_2_ in the presence of Ba(OH)_2_ and RbCO_3_ catalysts. Bio-oil yield enhanced by the addition of catalysts.

Electrochemical methods such as electrochemical degradation of lignin (Zhang et al., 2014), and electrolysis of corn stover to sugars (Xu et al., 2014) have also been reported. According to the literature, most of the electrochemical reactions were performed in ambient conditions. As novel approach, we developed a hybrid process, which basically combines hydrolysis and electrolysis in subcritical water to produce of high value chemicals from different types of biomass. Subcritical water has much lower dielectric constant than liquid water at ambient temperature. So, it was claimed that if constant current was applied to the reaction medium through specially designed electrodes in subcritical water environment, electrolysis could alter the hydrolysis reaction of cellulose in a way of protonation of intra-and inter-molecular hydrogen bonding around anode and as a result electrolysis in subcritical water could decrease necessary thermal energy to hydrolyze the β(1–4)glycosidic linkage. Therefore, we developed a green hybrid process by combining hydrolysis and electrolysis in subcritical water without using any toxic, organic solvents and catalyst. This hybrid process is applied on cellulose for the first time and synergetic effects of especially applied current and temperature on the product distribution and conversions of cellulose and total organic carbon (TOC) in the liquid product solution were revealed (Akin and Yuksel, 2016, 2017).

This green hybrid process named as hydrothermal electrolysis was conducted in a specially designed batch reactor (450 mL) in which cylindrical type titanium electrode was used as anode and reactor wall was behaved as cathode. In this research activity, effects of different parameters which are direct current, temperature, time and proton donor (sulfuric acid) concentration on the conversion of cellulose were investigated. The conversion of TOC and yields of products (5-HMF, levulinic acid, and furfural) were determined.

After obtaining the optimum conditions, it was proposed when constant voltage is applied, the degradation mechanism of cellulose and hydrolysis product distribution can selectively change. Constant voltage ranges from 4.0 to 8.0 V was applied between anode and cathode. 25 mM of H_2_SO_4_ was used as an electrolyte and also be a proton source. Hydrothermal electrolysis products of cellulose were investigated. After series of experiments, levulinic acid, 5-HMF and furfural was determined as the main decomposition products of cellulose by hydrothermal electrolysis. Detailed ionic and radical mechanisms of cellulose under constant voltage were revealed.

## Materials and Methods

### Chemicals

All chemicals used in experiments were at analytical grade and all solutions were prepared using de-ionized water. Microcrystalline cellulose (MCC) was purchased from Sigma Aldrich. The standards of reagents used in HPLC analysis are fructose (≥99%), levulinic acid (98%), lactic acid (98%), glycerolaldehyde (99%), glycolaldehyde (99%), 5-HMF (99%), glycolic acid (99%), and pyruvic acid (98%) and they were purchased from Merck. Sulfuric acid (96–98%) was also obtained from Merck. Phosphoric acid (85–90%) were purchased from Fluka. Biomass feedstock, hazelnut shell, was supplied from Ordu, Turkey. Hazelnut shells were dried in an oven at 60°C and then, they ground into small pieces (~1 mm) using a laboratory type grinder.

### Product Analysis

Elemental analysis of hazelnut shell was performed via CHNS-932, Leco, USA branded elemental analyzer. Moisture and ash contents of hazelnut shell (biomass) were determined by Thermal Gravimetric Analysis (TGA-51, Shimadzu, Japan). Hazelnut shell contains cellulose, hemicellulose and lignin and their amount were determined by Van Soest Method (Gozaydin and Yuksel, 2017).

The concentration of liquid products was determined via High Performance Liquid Chromatography (HPLC) and Gas Chromatography with Mass Spectroscopy (GC–MS, Agilent 6890 N/5973 N Network) were used to identify the liquid products which were unidentified in HPLC analyses. In HPLC analysis, sugar column (Shodex, SH1100) was used to separate products clearly and the temperature of column was 40°C. 3.75 mM H_2_SO_4_ was used as eluent and its flow rate was 0.5 ml/min. Refractive index (RID) was used for detection of aldehydes and organic acids. TOC conversions in the liquid products were determined by TOC analyzer (Shimadzu TOC-VCPH).

### Experimental Procedure

Experiments related to liquefaction of cellulose and hazelnut shell waste were performed in a Parr Instrument Company, USA branded 316 stainless steel autoclave (300 mL). The reactor equipped with gas inlet and outlet valves, liquid sampling valve, pressure gage, safety rupture disc, magnetic-driven impeller, internal thermocouple, and cooling loop (Gozaydin and Yuksel, 2017). Firstly, 4 g of biomass feedstock (cellulose, hazelnut shell waste) was placed into the reactor and then, 100 ml of de-ionized water was added to adjust the volume. After all the pins of reactor had been tightened, in order to remove the air inside the autoclave, the reactor was purged with nitrogen. Then, the reaction temperature was set to the desired reaction temperature with stirring rate of 200–250 rpm throughout the experiment. Temperature and pressures were recorded at every 5 min until the temperature reached the desired reaction temperature. The temperature and internal pressure rose until reaching desired reaction temperature and then, the reaction was started for a certain reaction time. At the end of reaction time, the heater was turned off and the system was cooled to 40°C to collect the final products (Gozaydin and Yuksel, 2017).

**Figure 2:**
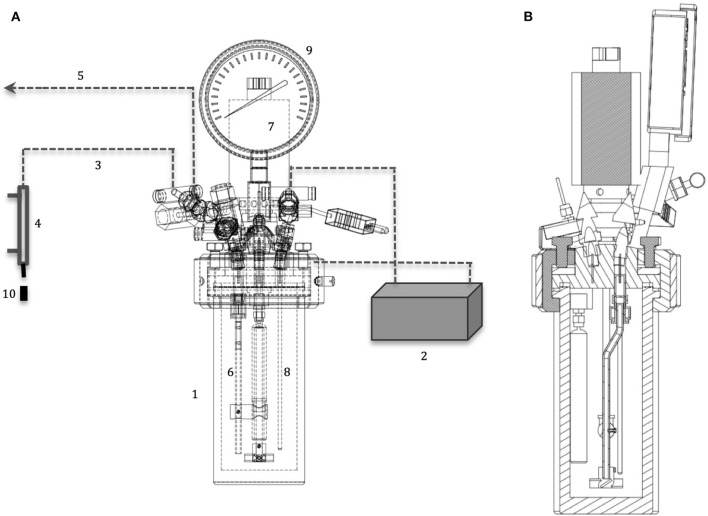
**(A)** Auxiliary view of hydrothermal electrolysis reactor: (1) electrical heater, (2) dc power controller, (3) liquid sampling tubing, (4) heat exchanger, (5) gas sample collector, (6) anode, (7) rotor, (8) thermocouple, (9) pressure gauge, (10) sample holder; **(B)** section view of hydrothermal electrolysis reactor.

Hydrothermal electrolysis experiments were performed in a 450 mL of SUS 316 stainless steel batch reactor (Parr 5500 series) with specially designed anode (titanium electrode) and it is illustrated in Figure 2.

Hydrothermal electrolysis experiments were carried out at a constant current (0–2 A) passing through the electrodes. Specially designed cylindrical type titanium electrode (D: 12 mm, L: 94 mm) was used as anode and cylindrical reactor wall (D_o_: 76 mm, L: 165 mm) was acted as cathode. The experiments were carried out to understand the reaction pathway by using H_2_SO_4_ solution (25 mM) at 200°C and applying constant voltage (0, 4.0, and 8.0 V) for 180 min. Liquid samples were collected in every 30 min. after the desired reaction temperature was reached. Collected liquid samples were filtered and analyzed immediately without any further treatment (Akin and Yuksel, 2016).

## Results and Discussion

### Liquefaction of Cellulose and Waste Hazelnut Shell

**Figure 3:**
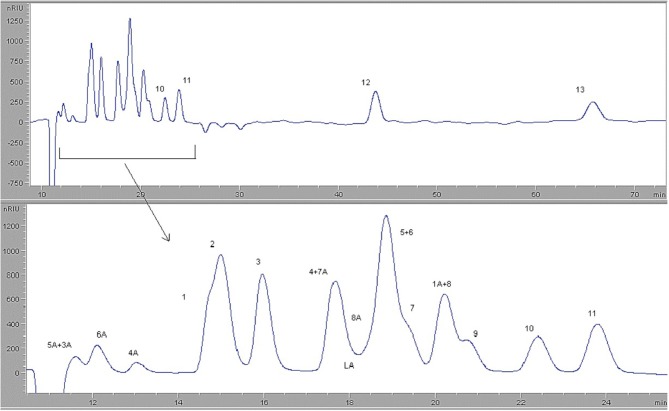
HPLC chromatograms of standard compounds at column temperature of 40°C: (1) pyruvic acid, (2) glucose, (3) fructose, (4) DL-glyceraldehyde, (5) glycolic acid, (6) glycolaldehyde, (7) lactic acid, (8) glycerol, (9) formic acid, (10) acetic acid, (11) levulinic acid, (12) 5-HMF, (13) furfural.

During this section, in order to investigate the effect of different parameters which are reaction temperature, initial pressure, reaction time, external oxidant concentration, the experiments were performed at 150, 200, 250, and 280°C and 0, 5, 10, and 15 bars for 30, 60, 90, and 120 min using 0, 5, 25, 50, 75, 100, and 125 mM H_2_O_2_ as an external oxidant. After degradation of microcrystalline cellulose with H_2_SO_4_ in hot-compressed water, liquid product consists of pyruvic acid, glycolaldehyde, glyceraldehyde, formic acid, glycolic acid, lactic acid, acetic acid, levulinic acid, 5-HMF, glycerol, and furfural was formed. Besides, in liquid product, some oligomers such as cellobiose, cellotriose, etc. and monomers such as glucose, fructose were also formed and they were identified HPLC analysis. HPLC analysis conditions were mentioned previously and HPLC chromatograms of the standard solutions are given in Figure 3.

According to the result of TOC analysis, the initial carbon amount of cellulose was determined as 42–45%. The yield of product % was determined using this data and the carbon number of products and the relation is given in Equation 1. Carbon balance was found to be more than 90% in all experiments. The conversion of cellulose and product selectivity were calculated using Equations (2) and (3), respectively.

(1) $$\begin{array}{r} \text{Yield of Product }\%=\frac{\left(\text{Number of carbon of species}\right)*\left(\text{Mole of species produced}\right)}{\text{Moles of carbon in cellulose}}*100 \end{array}$$

(2) $$\begin{array}{r} \text{Converted Cellulose }\%\;=\;\frac{Initial\;amount\;of\;cellulose\;\left(gram\right)-Residual\;amount\;at\;the\;end\;\left(gram\right)}{Initial\;amount\;of\;cellulose\;\left(gram\right)}*100 \end{array}$$

(3) $$\begin{array}{r} \text{Selectivity }\%=\frac{\left(\text{Number of carbon of species}\right)*\left(\text{Mole of species produced}\right)}{\text{Total Moles of carbon }\left(\text{TOC}\right)\text{ in liquid}}*100 \end{array}$$

**Figure 4:**
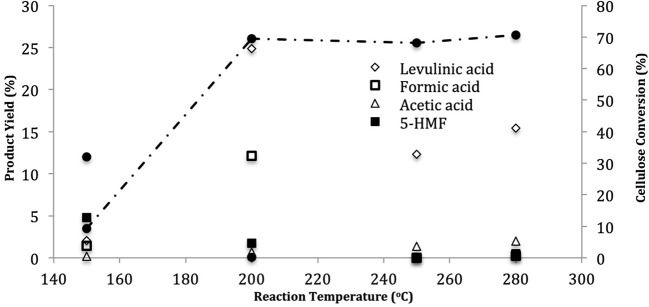
Conversion of cellulose and distribution of the liquid products after 60 min with 50 mM H_2_SO_4_ at different reaction temperatures (pressure range: 5–64 bar).

Cellulose has glycosic bonds so that is show high chemical resistance. In order to break these bonds, subcritical water can be used. Hence, usage of subcritical water provides a favorable environment and reaction medium for degradation of cellulose to desired product (levulinic acid) (Cardenas-Toro et al., 2014). After breakdown of glycosic bonds, de-polymerization of cellulose is occurred and hence, oligosaccharides and monosaccharides (glucose) can formed. After that, hydrolysis and rearrangement/decomposition of glucose and other monomer sugars is occurred and thus, fructose, 5-HMF, furfural, pyruvaldehyde, glyceraldehyde, and glycolaldehyde are formed. Furthermore, hemicellulose is hydrolyzed into oligomers (low and high molecular weight), xylose, furfural, glycolaldehyde, and glyceraldehydes. At higher reaction temperatures, re-polymerization, isomerization and fragmentation of them take place in liquid, gaseous and solid products (Cardenas-Toro et al., 2014). Additionally, organic acids (levulinic acid (desired product), acetic acid, formic acid, etc.) are also formed. Cellulose conversion (%) and product yields (%) were calculated for different reaction temperatures and the results are given in Figure 4.

As seen in Figure 4, the degradation of cellulose was observed as 70% after 60 min at 280°C. The conversion of cellulose is very low when temperature and pressure 150°C and 5 bar, respectively. At this condition, initial amount of cellulose was 4 g and the remaining amount was 3.61 g. It could be concluded that this reaction temperature (150°C) and pressure (5 bar) was not enough to breakdown of glicosidic bonds of cellulose. However, when temperature was 200°C, whereas yield of desired product (levulinic acid) and degradation of cellulose was suddenly enhanced, the amount of 5-HMF in liquid product decreased to 1.7% (Yüksel, 2016). Thus, it could be concluded that cellulose could be degraded under hydrothermal conditions through two steps so liquid products were formed. Glucose isomerized to fructose at higher reaction temperatures and then, fructose converted to the main intermediate which is 5-HMF (Rosatella et al., 2011). After that, this intermediate leads to form various final compounds. One of the final products is levulinic acid and by-product of it is formic acid which is formed at higher reaction temperatures (Zeng et al., 2010; Rosatella et al., 2011; Cardenas-Toro et al., 2014). This obtained data from literature is matching with our findings. As shown in Figure 4, under the conditions with low concentrations of levulinic acid and formic acid were obtained, 5-HMF yield was low that shows the dehydration of 5-HMF. According to Kruse and Gawlik (2003), one of the minor organic acids formed from the decomposition of cellulose in subcritical water was acetic acid. In Kruse's study, the concentration of acetic acid was not significantly affected by temperature which is consistent with the results shown in Figure 4.

**Figure 5:**
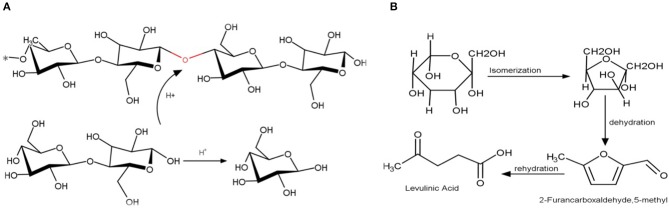
**(A)** Cellulose hydrolysis to cellobiose and glucose; **(B)** glucose isomerization to fructose, dehydration and rehydration to 5-HMF and levulinic acid.

The reaction pathway of cellulose can be separated to three main parts: the first part is about to the hydrolysis of cellulose to oligosaccharides; the second part is related with the hydrolysis of saccharides to glucose (Figure 5) and; the last part comprises the glucose isomerization and dehydration to 5-HMF and levulinic acid. In the first part, oligosaccharides and small saccharides such as cellobiose are formed as the products of cellulose hydrolysis (Cantero et al., 2013).

The effect of temperature on levulinic acid (desired product) yield at different reaction times was investigated and the results are given in Table 1. When reaction was carried out at 200°C, whereas levulinic acid was not detected in the liquid product solution during the first 30 min, levulinic acid yield reached to 24.9% at the end of 60 min. Levulinic acid yield raised to 25.2% in the first 30 min when the reaction is carried out at higher reaction temperatures (250°C). However, yield of levulinic acid has decreased to 14.3% for longer reaction times up to 120 min. The opposite case was observed for lower reaction temperature such as 150°C. The yield of levulinic acid increased from 1.5 to 6.1% when the reaction was prolonged, however, it was still lower than the levulinic acid yield at 200°C (Yüksel, 2016). According to Girisuta (2007), the highest levulinic acid yield was obtained at higher reaction temperature in a shorter time. At the same study, it was reported that under the conditions at which high levulinic acid yield was obtained, 5-HMF yield was low in the liquid product solution.

**Table 1:** Levulinic acid yield with 50 mM H_2_SO_4_ from 30 to 120 min at various reaction temperatures of 150, 200, 250, and 280°C.

**Temperature (**^****°****^**C)**				
**Time (min)**	**150**	**200**	**250**	**280**
30	1.49	0.10	25.19	16.76
60	2.04	24.90	12.33	15.42
90	2.40	19.28	18.40	18.69
120	6.07	17.94	14.32	16.20

To comprehend the effect of H_2_SO_4_ concentration (5, 25, 75, 100, and 125 mM) on the product distribution was also investigated. In that case, yield of levulinic acid enhanced with respect to the increasing H_2_SO_4_ concentration. While the lower yield of levulinic acid was observed as 4.8% using 5 mM H_2_SO_4_), the highest yield was observed as 38% with the addition of 125 mM H_2_SO_4_. Similar trend was observed in the yield of formic acid. The yield of formic acid was observed as 2.6 and 21% using 5 and 125 mM H_2_SO_4_, respectively (Yüksel, 2016). However, the yields of glucose, fructose, and 5-HMF were started to decrease with respect to the increasing acid concentration in the feed and the reaction mechanism of cellulose degradation in hot-compressed water could be lead to decrease in yield of these products. In similar studies in literature, it was found that firstly, cellulose degraded to simple sugars (glucose and fructose). Then, main intermediate (5-HMF) was formed by the conversion of them. Finally, levulinic acid, and formic acid were formed by the decomposition of 5-HMF (Zeng et al., 2010; Rosatella et al., 2011; Cardenas-Toro et al., 2014).

After investigating cellulose behavior in sub-critical water, we used hazelnut shell waste as real lignocellulosic biomass source. Cellulose, hemicellulose and lignin contents of hazelnut shell (Table 2) were found by using Van Soest Method (Goering and Van Soest, 1970; Madenoglu et al., 2014).

**Table 2:** Structural carbohydrate analysis of hazelnut shell.

**Ultimate analysis (wt%)**	
C	50.44
H	6.76
N	0.76
S	0.11
O	41.92
**Proximate analysis (wt%)**	
Moisture	8.93
Ash	1.48
Protein	3.11
**Structural analysis (wt%)**	
Cellulose	36.02
Hemicellulose	12.66
Lignin	40.14
Extractives	7.86

During the degradation of cellulose in subcritical water, firstly, the dominant mechanism is de-polymerization of cellulose and hence, oligosaccharides, and monosaccharides were formed. After that, hydrolysis and rearrangement/decomposition of glucose and other monomer sugars occur so that various products such as 5-HMF, glyceraldehyde, fructose, furfural, pyruvaldehyde, and glycolaldehyde could form. Besides, hydrolysis of hemicellulose take place to form low and high molecular weight oligomers, xylose, glycolaldehyde, furfural, and glyceraldehydes and the re-polymerization, isomerization and fragmentation of them happen in solid, liquid and gaseous products when the reaction temperature is high (Pavlovic et al., 2013; Cardenas-Toro et al., 2014). Various compounds were formed in liquid product, however, the production of levulinic acid, acetic acid, 5-HMF and furfural had great attention.

Maximum hazelnut shell conversion was found as 65.4% at the following conditions: 280°C, 120 min, 50 mM H_2_SO_4_. When the reaction temperature increased from 150 to 280°C, hazelnut shell conversion increased considerably. Besides, TOC conversion was also determined solid residues and the results are given in Table 3. TOC conversion was found as 49.71% at 280°C and 60 min (Gozaydin and Yuksel, 2017).

**Table 3:** The effect of reaction temperature on TOC conversion at different reaction temperature with addition of 50 mM H_2_SO_4_ concentration.

**Temperature (^**°**^C)**	**Time (min)**	**TOC Conversion (%)**
150	15	29.59
	60	38.49
	90	35.01
	120	32.61
200	15	47.73
	60	45.87
	90	46.08
	120	46.96
250	15	46.52
	60	47.30
	90	45.04
	120	47.41
280	15	43.39
	60	49.71
	90	48.26
	120	47.92

In literature (Girisuta, 2007; Toor et al., 2011) various types of acids were described to decompose different biomasses in subcritical water. In our study, as an oxidizing agent sulfuric acid was used. As it seen in Table 4, the formation of levulinic acid was not observed for shorter reaction times when the sulfuric acid was not used. However, the degradation of hazelnut shell to form levulinic acid enhanced by the addition of H_2_SO_4_ for the same reaction conditions. In the light of the studies of Efremov et al. (1997) and Fang and Hanna (2002), it could be deduced that levulinic acid yield enhanced with respect to the increasing amount of sulfuric acid. The product yields in our study show the same behavior in line with the literature. Moreover, while the addition of sulfuric acid causes an increase in levulinic acid yield (up to 11.91%) at 150°C, it did not affect at 280°C.

**Table 4:** The yield of levulinic acid, furfural, and acetic acid in presence and absence of sulfuric acid (50 mM) at different reaction time (150 and 280°C).

**Time (min)**	**150^**°**^C without acid**	**280^**°**^C without acid**	**150^**°**^C with acid**	**280^**°**^C with acid**
**LEVULINIC ACID YIELD (%)**				
15	0	0.40	0	8.42
60	0	0.89	0	11.85
90	0	1.22	0.10	13.26
120	2.17	1.05	0.28	13.05
**FURFURAL YIELD (%)**				
15	0	1.85	1.54	0.25
60	0.08	0.76	5.54	0.18
90	0.389	0.40	6.60	0.07
120	0.77	0.23	11.91	0.04
**ACETIC ACID YIELD (%)**				
15	1.17	9.30	11.58	8.28
60	2.07	10.64	9.53	8.09
90	1.80	9.51	11.09	8.78
120	1.94	8.65	10.97	8.76

**Figure 6:**
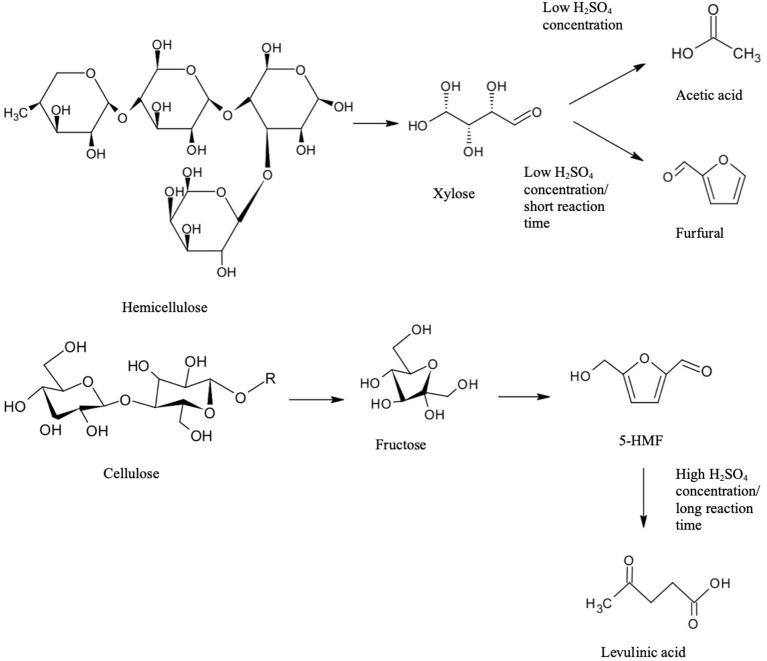
Main proposed reaction pathways for hydrothermal conversion of waste hazelnut shell.

Main reaction mechanisms of the liquefaction of waste hazelnut shell were purposed based on those results, and they were illustrated in Figure 6. Under subcritical water media, the rate of cellulose hydrolization to oligomers and isomerization of glucose into fructose were rapid at low temperature. The rate of rehydration of 5-HMF into levulinic acid increased when H_2_SO_4_ (100–125 mM) was used excessively and long reaction time (≥60 min). Two main reaction pathways were possible for hemicellulose. When hydrothermal reaction was performed at low H_2_SO_4_ concentration (0–5 mM) at 200°C, acetic acid formation was favored by the cleavage of acetyl (Zhu et al., 2014).

### Hydrothermal Electrolysis of Cellulose

Hydrothermal electrolysis experiments were carried out in a 450 mL of SUS 316 stainless steel batch reactor (Parr 5500 series), which is illustrated in Figure 2. All experiments were carried out using deionized water (200 ml) and cellulose (8 gr). Sulphuric acid was used as an external proton source and it was added to the reaction medium at different concentrations (1–50 mM). Reaction time started when the reaction medium had reached the desired reaction temperature and the first sample (2 mL) was taken for reaction times of 0 and then, other samples were taken at the reaction times of 15, 30, 60, 90, and 120 min. So that, the effect of heating period was investigated.

Experiments of hydrothermal electrolysis were performed at a constant current which are 0 and 2 A passing through the electrodes. In this set-up, anode was specially designed cylindrical type titanium electrode (D: 12 mm, L: 94 mm), cylindrical reactor wall (D_o_: 76 mm, L: 165 mm) was used instead of cathode. To purpose the reaction pathway of degradation of cellulose when current was applied, the reaction conditions were as follows: 25 mM H_2_SO_4_ solution at 200°C with application of constant voltages (0, 4.0, and 8.0 V) for 180 min.

Sub- and supercritical water have a great attention as a reaction medium because of its versatile properties. For example, this reaction medium has 10^−11^ of ionic product concentration (200–300°C range) and dielectric constant is ≈10 (near critical) (Marshall and Franck, 1981). Water can self-dissociated into hydroxide and hydronium ion and these ions have a critical role for the protonation of β(1–4)glycosidic bond of cellulose so that cellulose could be decomposed. If hydronium ion must access to intra- and inter-molecular hydrogen bonds then, microcrystalline cellulose could be depolymerized. Hence, diffusion of protons with versatile properties such as high diffusivity and low density of near critical water becomes more effective. When ionic product concentration is high, low energy is needed for the migration of electroactive species in electrochemical methods. Thus, electrochemical reactions at near critical conditions become more economically feasible (Asghari and Yoshida, 2008).

**Figure 7:**
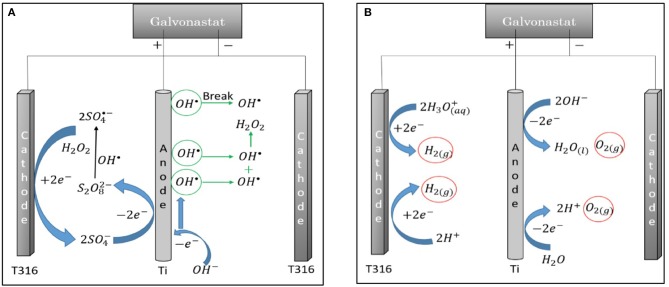
Electrochemical reaction of water and sulfuric acid that yields **(A)** radical, **(B)** ionic products.

The hypothesis of this is that application of direct current into reaction medium under hydrothermal condition would create activated species in terms of ionic and radical molecules due the redox reactions of water and sulfuric acid. Formation of these molecules can alter the decomposition of cellulose as in the postulated reaction mechanism (Figure 7). Application of direct current under hydrothermal condition is resulted in the reactions at positive anode (Equations 4, 5) and at negative cathode (Equations 6, 7) Equation (5) yield the formation of H^+^ ion which can generate an acid layer around anode that can yield the protonation of β(1–4)glycosidic bond of cellulose.

(4) $$\begin{array}{r} {\;4OH}_{\left(aq\right)}^{-}-4e^{-}\to {2H}_{2}O_{\left(l\right)}+O_{2\left(g\right)} \end{array}$$

(5) $$\begin{array}{r} 2H_{2}O_{\left(l\right)}-{4e}^{-}\to {4H^{+}}_{\left(aq\right)}+O_{2\left(g\right)} \end{array}$$

(6) $$\begin{array}{r} {4H}_{\left(aq\right)}^{+}+{4e}^{-}\to 2H_{2\left(g\right)} \end{array}$$

(7) $$\begin{array}{r} {4H_{3}O}_{\left(aq\right)}^{+}+{4e}^{-}\to {2H}_{2\left(g\right)}+{4H}_{2}O_{\left(l\right)} \end{array}$$

A hybrid method comprised of electrochemical oxidation and the production of value added organic species in sub-critical water has gained attraction (Yuksel et al., 2011). Our previous study is related with the decomposition of microcrystalline cellulose in sub-critical water conditions where the reaction temperature varied between 170 and 230°C in the presence of sulfuric acid (1–50 mM) by applying direct constant current (0–2 A). Fractional factorial experimental design was performed to determine the coupled and interaction parameters of constant current with sulfuric acid under hydrothermal conditions. According to the results, when 50 mM H_2_SO_4_ was used and 1 A current was applied to the reaction medium, the required reaction temperature for the maximum cellulose conversion of 82% decreased from 230 to 200°C (Akin and Yuksel, 2016). The application of direct current provides the formation of radicals ($OH^{.-},SO_{4}^{.-}$) and ionic products (*OH^-^,H_3_O^+^*), so that TOC conversion enhanced. As current values increased, concentration of radicals in the reaction medium increased. However, TOC conversion decreased when higher current (2 A) was applied. Additionally, the application of direct current (1–2 A) affected the formation yields of 5-HMF and levulinic acid. While the yield of 5-HMF enhanced by direct current, levulinic acid concentration decreased. During the reaction, variable electrode potential was observed because of the application of constant current to reaction medium. Hence, the change in electrode potentials leads to formation of various activated species within the reaction medium. Davis et al. (2014) carried out a study over electrolysis of sulfuric acid solution to form persulfate based on radical mechanism. This study show that the formation of different radical species such as $SO_{4}^{-•},HSO_{4}^{•},OH^{•}$ and ionic products such as $SO_{4}^{2-},H_{3}O^{+},OH^{-}$ can be observed during electrolysis of sulfuric acid solution at different electrode potentials (Davis et al., 2014). Formation of these products can alter the decomposition mechanism of cellulose to form levulinic acid, furfural and 5-HMF selectively.

Effect of applied voltage (4.0 and 8.0 V) on product selectivity and TOC conversion in hydrothermal reaction medium was investigated in the presence and absence of acid catalyst (H_2_SO_4_). Selectivity values of cellulose on formation of furfural, 5-HMF and levulinic acid were determined as described in material and methods section. The control reaction was carried out by applying voltage under hydrothermal conditions (200°C) in the absence of acid catalyst. Results are listed on Table 5.

**Table 5:** TOC Yield, conversion of cellulose, and valuable product selectivities at applied voltage values of 0, 4.0, 8.0 V at different electrolyte (H_2_SO_4_) concentrations as 0 and 25 mM.

**No acid**					**With 25 mM acid**		
**In %**	**Voltage (V)**	**0 min**	**60 min**	**180 min**	**0 min**	**60 min**	**180 min**
TOC yield	0	2.4	4.1	9.8	10.1	23.8	49.9
	4	2.2	2.6	7.7	9.4	28.7	45.4
	8	2.1	4.0	13.8	10.3	24.6	50.3
Conversion of cellulose	0		9.4	11.3		48.7	77.1
	8		70.6	78.2		30.7	82.0
Levulinic acid selectivity	0	4.3	6.3	1.4	12.4	12.8	10.3
	4	0.2	0.3	0	9.4	10.8	19.1
	8	0.2	0	0	8.8	9.2	23.7
5-HMF selectivity	0	0.8	1.4	9.7	2.7	42.8	26.1
	4	6.9	2.1	3.3	8.1	29.9	21.8
	8	3.9	4.8	15.9	2.2	17.9	9.1
Furfural selectivity	0	7.1	3.7	5.3	2.9	9.6	5.8
	4	2.8	3.2	6.1	6.2	7.6	5.1
	8	2.7	11.3	14.3	5.9	6.3	3.7

When cellulose was degraded under hydrothermal conditions (no current, no electrolyte), TOC was recorded as 13.8% (Table 5). Whereas, lower TOC conversions were observed by applying constant voltage (4.0 V) compared to current free experiments, higher TOC values were observed at constant voltage (8.0 V). Rossmeisl et al. (2005) showed that higher potentials could cause to dissociate the water into its ionic products and the formation of radical species such as hydroxyl radical (OH^•−^) could enhance under hydrothermal conditions by applying voltage. Hence, in the light of literature, it is possible to say that higher TOC conversions could be achieved by higher voltage. If concentration of radical species increases, decomposition products could form via radical based mechanism. The coupled effect sulphuric acid (25 mM) and applied voltage can be explained by the oxidation of sulphuric acid to form the sulfate radical (SO^•^_4_^−^) near anode (Davis et al., 2014). This synergetic effect causes an increase in the TOC yield to 50.3% (Table 5) under hydrothermal conditions (Akin and Yuksel, 2016).

The conversion of fructose to furfural under hydrothermal conditions could be proposed by two reaction pathways (Aida et al., 2007). First one is the removal of formaldehyde from 5-HMF and the second one is the cleavage of C–C bond in fructose so that pentose formation is observed and then dehydration of pentose leads to formation of furfural (Luijkx et al., 1993). The selectivity values of furfural and 5-HMF increased simultaneously. Hence, it could be concluded that furfural might be formed by the cleavage of C–C bond in fructose due to the low yield of fructose (8.0 V) in comparison to hydrothermal reaction (0 V). Rehydration of 5-HMF resulted in formation of levulinic acid (Akin and Yuksel, 2017).

**Figure 8:**
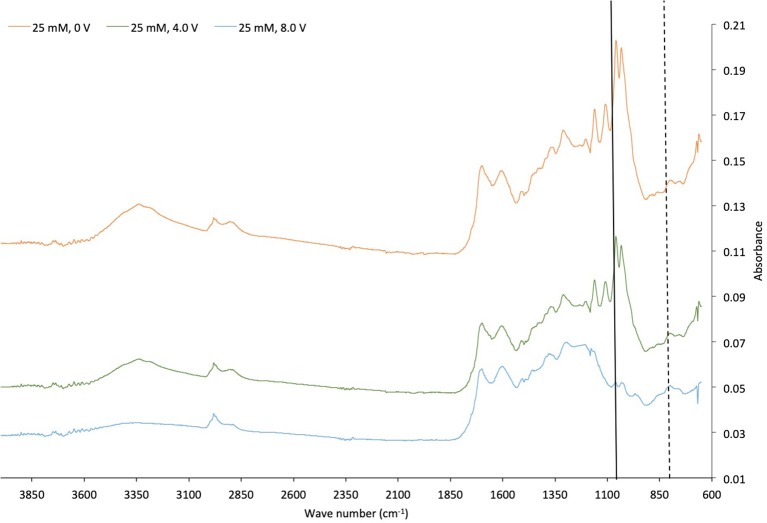
FTIR spectrum of solid residues of applied potential of 0, 4.0, and 8.0 V in 25 mM sulfuric acid medium.

As seen on Table 5, in the first 60 min, selectivity of 5-HMF was 42.8% and further reaction time selectivity of levulinic acid was 23.7%. FTIR results (Figure 8) showed that S=O Sulfoxide (solid line in Figure 8) and S-OR Ester (dashed line in Figure 8) groups were formed at solid residuals when voltage (4.0 and 8.0 V) was applied. The activity of sulfonated carbon particles might be hindered due to the high concentrations of sulfone ion radicals which is a reactive species formed at relatively high concentration of sulphuric acid by applying potential (Akin and Yuksel, 2017).

From Table 6, it was clearly seen that, when 2 A current was applied for 120 min., there was a dramatic increase in H_2_ concentration from 0.5 to 75.3 μg/ml. Under same conditions, CO_2_ concentration decreased from 530.5 to 398.7 μg/ml. Under the effect of applied current with the addition of 50 mM H_2_SO_4_, H_2_ concentration increased from 75.3 to 98.2 μg/ml, whereas CO concentration was recorded as 26.2 to 48.7 μg/ml, respectively. This result indicated that water gas shift reaction was favored the carbon monoxide formation with the addition of sulfuric acid as an electrolyte. Methane formation was also observed as gas products and an increase in acid concentration from 0 to 50 mM yields to 0.26 and 0.53 μg/ml, respectively.

**Table 6:** Gaseous products (H_2_, CO, CH_4_, CO_2_) of cellulose of degradation under hydrothermal and applied constant current conditions at 230°C and 120 min.

		**Concentration of gas products (μg/ml)**			
**H**_**2**_**SO**_**4**_ **(mM)**	**Current (A)**	**H**_**2**_	**CO**	**CH**_**4**_	**CO**_**2**_
0	0	0.5	52.4	0.21	530.5
0	2	75.3	26.2	0.26	398.7
50	2	98.2	48.7	0.53	418.7

**Figure 9:**
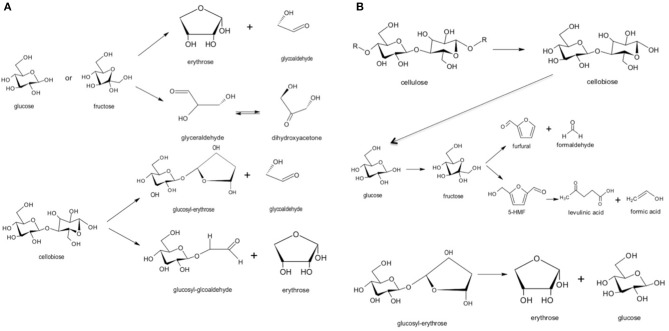
Proposed **(A)** radical and **(B)** ionic reaction pathways for hydrothermal electrolysis of cellulose at constant voltage (8.0 V) with 25 mM H_2_SO_4_.

The application of constant voltage lead to form radicals and ionic products so that levulinic acid, furfural and 5-HMF can be formed from cellulose selectively. Although it is hard to distinguish the radical and ionic-based mechanism due to common product distribution, under certain potential in the designed hybrid reactor system favors the degradation of cellulose via radical mechanism (Akin and Yuksel, 2019). Proposed ionic and radical reaction pathways for hydrothermal electrolysis of cellulose at constant voltage are given in Figure 9.

As the electrode potential (8.0 V) was applied, various activated radical species such as SO_−4_^•^, HSO^•^_4_, OH^•^ and ionic species such as ${\text{SO}}_{4}^{2-}$, ${\text{H}}_{3}^{-}$O^+^, OH^−^ could commonly be created when electrolysis was carried out in the presence of H_2_SO_4_ in sub-critical water. Formation of these products can shift degradation mechanism of cellulose to high value chemicals such as levulinic acid, furfural and 5-HMF (Figure 9). Additionally, formation of glucosyl-glycoaldehyde, glucosyl-eryhtrose, and glycoaldehyde was also observed when hydrothermal electrolysis was carried out at 8.0 V so that decomposition mechanism of cellulose via ionic and radical based pathways was revealed (Figure 9).

## Conclusion

Hydrolysis of cellulose in sub-critical water conditions has been widely searched because of the versatile properties (low dielectric constant, high diffusivity, high ion product concentration etc.) of hot compressed water as reaction medium. Thus, high ionic product concentration and its high diffusivity to intra and inter molecular hydrogen bonds enhanced the protonation of β(1–4) glycosidic linkage of cellulose that yield degradation products which are glucose, fructose, 5-HMF, furfural, and levulinic acid.

As model biomass, hazelnut shell waste was converted to value-added chemicals under hot compressed water. In the context of experimental study, the effect of reaction temperature, reaction time and acid (H_2_SO_4_) concentration was investigated. The main products of hydrothermal acid-treatment were clarified as levulinic acid, furfural and acetic acid by HPLC analysis. As reaction temperature increases, a considerable improvement on the amount of formed levulinic acid and conversion of hazelnut shell was observed. For instance, when the reaction temperature, time and acid concentration were 280°C, 120 min and 50 Mm, respectively, levulinic acid yield and conversion of hazelnut shell were found as 13.05 and 65.40%, respectively. Addition of H_2_SO_4_ enhanced the production of levulinic acid from waste hazelnut shell.

As alternative novel method to frequently used ones, a novel hybrid system for the conversion of cellulose to high value chemicals was introduced. The application of direct current in constant mode was investigated and was found that applied 1 A of current resulted in higher total organic carbon (TOC) in comparison to current free experiments. The enhancement in TOC was conducted for the formation of an acid layer around anode due to the self-dissociation of water that hydrolyses cellulose. It was proposed that application of constant voltage could selectively change the degradation mechanism of cellulose and hydrolysis product distribution. The detailed product distribution of decomposition of microcrystalline cellulose under subcritical water condition by application of direct current was investigated and reaction pathway for hydrothermal electrolysis of cellulose was proposed.

## Data Availability Statement

All datasets generated for this study are included in the article/supplementary material.

## Author Contributions

The author confirms being the sole contributor of this work and has approved it for publication.

### Conflict of Interest

The author declares that the research was conducted in the absence of any commercial or financial relationships that could be construed as a potential conflict of interest.
